# Effect of Cellulase Enzyme Produced from *Penicillium* *chrysogenum* on the Milk Production, Composition, Amino Acid, and Fatty Acid Profiles of Egyptian Buffaloes Fed a High-Forage Diet

**DOI:** 10.3390/ani11113066

**Published:** 2021-10-27

**Authors:** Hossam H. Azzaz, Ahmed M. Abd El Tawab, Mostafa S. A. Khattab, Małgorzata Szumacher-Strabel, Adam Cieślak, Hussein A. Murad, Maciej Kiełbowicz, Mohamed El-Sherbiny

**Affiliations:** 1 Department of Dairy Science, National Research Centre, 33 Bohouth St., Dokki, Giza 12622, Egypt; hosam19583@gmail.com (H.H.A.); amaeid2010@gmail.com (A.M.A.E.T.); msakhattab@gmail.com (M.S.A.K.); murad951@hotmail.com (H.A.M.); 2 Department of Animal Nutrition, Poznań University of Life Sciences, Wołyńska 33, 60-637 Poznań, Poland; malgorzata.szumacher@up.poznan.pl (M.S.-S.); adam.cieslak@up.poznan.pl (A.C.); 3 Department of Internal Diseases and Diagnostics, Poznan University of Life Sciences, Wołyńska 35, 60-637 Poznań, Poland; maciej.kielbowicz@up.poznan.pl

**Keywords:** buffaloes, cellulase, exogenous enzymes, nutrient intake, milk, fatty acid

## Abstract

**Simple Summary:**

Exogenous fibrolytic enzymes can improve nutrient digestibility of feeds high in fibrous content offered to Egyptian lactating buffaloes. The proposed cellulase exclusively produced in-farm using *Penicillium Chrysogenum* showed higher activity in previous in vitro studies. That is why it was chosen to get tested against a well-known commercial source of cellulase enzyme from the Egyptian markets for its efficiency in increasing milk productivity and composition. Profiles of amino acids and fatty acids were also recorded. The initial results highlighted a superiority of the produced enzyme (FENZ) against the commercial source (CENZ). It was also clear that FENZ can preserve higher proportions of fatty acids in the milk, primarily conjugated linoleic acid. Based on the idea rationale, our conclusion is to promote setting a small cellulase production unit in each farm in Egypt to decrease the cost of feeding by using agricultural and agro-industrial waste during the cellulase production and feeding process.

**Abstract:**

The experiment was conducted to study the effects of supplementing a cellulase enzymes cocktail to lactating buffaloes’ diet, on the nutrient intake, nutrient digestibility, and milk production performance and composition. Twenty-four lactating Egyptian buffaloes were assigned into one of the following treatments: CON—control consisted of a total mixed ration, CENZ—the total mixed ration supplemented by a commercial source of cellulase enzyme, FENZ—the total mixed ration supplemented with cellulase enzyme cocktail produced in-farm. Supplementing the diet with the in-farm source of cellulase (FENZ) had a significantly higher impact on crude protein, neutral detergent fiber, and acid detergent fiber digestibility. However, FENZ tended to increase the EE digestibility compared to CENZ. FENZ showed significantly higher nutrient digestibility percentages compared to other groups. Supplementing the diet with cellulase enzymes (CON vs. ENZ) significantly increased the daily milk yield and the fat correct milk yield; both yields were significantly higher with FENZ than all groups. Oleic, linoleic, and linolenic acid concentration were significantly higher with cellulase enzymes supplementation (CON vs. ENZ) and the conjugated linoleic acid concentration. Supplementing fungal cellulase enzyme produced on a farm-scale has improved milk productivity, fat yield, and milk fat unsaturated fatty acids profile in lactating buffaloes.

## 1. Introduction

Ruminant-based agriculture relies on several key processes to reach food security worldwide; one of those keys is the ability to provide farm animals products (meat or milk) that cover the increased needs of rising populations, mainly by providing animal feed sources that do not compete with human crops land area [1,2]. Most ruminant feeds consist of forages with high fibrous content, mainly because they can degrade plant cell wall material by the rumen microorganisms and their associated enzymes. However, rumen digestion is not perfect, and usually a high amount of plant fiber bypasses the digestive tract without being used [3,4]. Plant biomass’s significant portion (up to 50% dry weight) consists of cell wall cellulose polymers. It is well known that cellulose, consisting of β-1,4 glucosidic bonds linking D-glucose molecules, is by far the ultimate renewable resource naturally [5]. Recently, there has been a rising interest in using agro-industrial waste and agroforestry products in ruminant nutrition. Regrettably, those materials’ nutritional composition is unbalanced and requires the support of high-quality grains, legumes, or additives to meet the nutritional needs [2].

Additionally, the high number of cross-links within plant cell wall carbohydrates and lignin through the growing season decrease digestibility and bounds the utilization of forages by ruminants. Consequently, it will demand several cellulolytic enzymes to degenerate these materials effectively [6,7,8]. As an outcome, many strategies have been exploited to enhance fiber degradation in the rumen. One of those strategies is the application of exogenous fibrolytic enzymes. Enzymes as a feed additive have been used extensively in the last decades to improve the nutritive value of diets by enhancing fiber digestion and, respectively, increase productive ruminant performance [9]. Research has shown that anaerobic fungi categorized extensively in the digestive tracts of ruminants can utilize various carbohydrates and maintain an efficient glycosyl hydrolase system that hydrolyzes plant carbohydrates [6,10]. The mechanisms by which cellulases’ enzyme from anaerobic rumen bacteria digest cellulose are poorly defined. However, the cellulases’ network in fungi consists of three cellulases: (1) endoglucanases, which randomly hydrolyze the 1,4-β-glycosidic bonds within cellulose, producing oligosaccharides with reducing and non-reducing ends; (2) exoglucanases, which separate cellobiose units from their nonreducing ends, and (3) β-glucosidases, which hydrolyze cellobiose and low-molecular-weight cellodextrins, producing glucose [11,12,13]. It is well known that fungi are the main cellulase-producing microorganisms. The *Penicillium chrysogenum* is a well-known cellulase producer with numerous commercially available products for agricultural and industrial uses. However, there is not enough knowledge on *Penicillium chrysogenum* produced cellulases on ruminant nutrition and performance [11,12].

On the other hand, agricultural and agro-industrial wastes like sugarcane bagasse, rice straw, wheat straw, orange peel, and palm fronds continually expand due to industrialization, and their disposal becomes a problem concerning area and creating environmental pollution. However, these wastes could serve as a cheap alternative source for microbial growth and biomass or enzymes production. This production system emerged as an appropriate technology for managing agro-industrial residues, with numerous advantages, including less pollution, high volumetric productivity, and a relatively greater concentration of products [14,15]. The current study aimed to investigate the effect of supplementing cellulase enzyme cocktail produced in-farm scale from *Penicillium Chrysogenum* to the diet of lactating buffaloes on the nutrient digestibility, milk production performance, milk amino acids, and fatty acids profile when compared to a commercial cellulase enzyme product from the Egyptian markets. We hypothesized that applying the in-farm enzyme cellulase will better impact the buffaloes’ digestibility, productivity, and milk fatty acid content due to the high activity of the produced fungal source of cellulases.

## 2. Materials and Methods

The experiment conducted in this study was carried out on Egyptian lactating buffaloes at ALSATAR farm in Khatatba City, Monufia Governorate, Egypt. The buffaloes were cared for following the animal research guidelines and ethics of the Ministry of Higher Education and Scientific Research, Egypt, and authorized by the local ethical committee of the National Research Centre, Egypt.

### 2.1. Experimental Design, Treatments, and Management

#### 2.1.1. Enzymes Supplements

Cellulolytic enzyme cocktail was produced in a small pilot plant prepared to set and prepared on the farmland for enzyme production using the methodology described earlier in [16]. Briefly, dried (70 °C for 24 h) and ground rice straw, wheat straw, and palm fronds were used as the primary substrate materials for the fungal cultures at 50%, 30%, and 20%, respectively. The fungal strain of *Penicillium chrysogenum* obtained from the Laboratory of Plant Pathology, National Research Centre, Cairo, Egypt, was used to produce the cellulase enzyme using the mentioned wastes as a source of cellulose. Briefly, 1000-mL conical flasks each containing 100 mL of cellulose powder medium that consisted of NaCl, 6 (g/L); (NH_4_)_2_SO_4_, 1 (g/L); K_2_HPO_4_, 1 (g/L); MgSO_4_.7H_2_O, 0.05 (g/L); CaCl_2_, 0.1 (g/L); yeast extract, 0.5 (g/L); Peptone, 0.5 (g/L); primary substrate powder, 4 (g/L). The medium pH was adjusted to 6 and then the growth cultures of fungi were employed as inoculant at rate of 5% (*v*/*v*) inoculum size, the fermentation then lasted three days at 30 °C. The fermented substrate for each flask was then mixed with 25 mL of 0.02 M acetate buffer (pH 5.0) to extract the enzyme, by shaking in a rotary shaker (120 rpm) for one hour at room temperature, that to extract the enzyme from the solid-state media. The activity of the extracted enzymes was measured in the obtained filtrate based on carboxymethyl-cellulase activity (CMC) and the reducing sugar liberated which was determined following the method of Dinitrosalicylic acid [16]. One cellulase unit is defined as the amount of enzyme that liberates reducing sugar at the rate of one µmol/mL/min under assay conditions. For comparison, a commercial source of cellulase enzyme (Pan-Zyme; BAYTARA for pharmaceuticals technology, Sadat Industrial City for Guangdong VTR Bio-Tech Co., Ltd., Guangdong, China) was introduced. Generally, the commercial each kilogram of Pan-Zyme had a cellulase activity of 21,081 International Units (IU). In comparison, the produced enzyme cellulase activity was 12,817 IU/kg. Both commercial and produced cellulase enzymes were supplemented with the buffaloes’ diet at 42.16 IU/Kg of dry matter following the recommendation of the commercial source of enzyme.

#### 2.1.2. Diet, Treatments, and Management

Twenty-four lactating Egyptian buffaloes (602 ± 15.2 kg body weight; 3.6 ± 0.55 parity, 65 ± 10 d in milk and 6 ± 1.1 kg/d of milk production; (mean ± SD)) were used in a complete randomized design with a 25-day adaptation period and a 5-day sampling period (total experimental period of 30 days). All buffaloes were initially housed in tie stalls and fed individually according to body weight to meet their requirements for lactation [17]. The buffaloes were then randomly allocated (8 buffaloes per group) to one of the following experimental groups: CON—control consisted of a total mixed ration as described in Table 1, CENZ—the total mixed ration supplemented by a commercial source of cellulase enzyme (42.16 IU/kg DM), FENZ—the total mixed ration supplemented with cellulase enzyme cocktail produced in-farm (42.16 IU/kg DM). Buffaloes were fed the total mixed ration (TMR) twice daily at 7 a.m. and 7 p.m. in equal portions 1 h before each milking (buffaloes were machine milked twice per day). The portions of TMR used for all treatments were prepared once every ten days to check the dry matter concentration and adjust the feed if necessary. The enzyme supplementation was applied to a small portion of the TMR offered individually for each animal twice per day before the morning and evening feeding to ensure that the animal received the specified amount of enzyme. Samples of fresh TMR were collected every ten days (during the TMR preparation) and throughout the sampling period and stored at −20 °C until analyzed chemically. The milk production was tracked daily and was only recorded during the sampling days. Milk samples were collected at each milking from all buffaloes throughout the sampling period. Morning milk samples were stored at 4 °C until the evening samples were collected. The samples were subsequently pooled according to morning and evening milk yield and prepared in two equal parts: one aliquot was immediately analyzed for fat, crude protein, and lactose by infrared analysis (Milkotester LM2, Belovo, Bulgaria), and the rest was stored at 20 °C for amino acid (AA) and fatty acid (FA) analysis. Milk total and non-fat solids content was calculated.

### 2.2. Sample Analysis

#### 2.2.1. Nutrient Intake and Digestibility

During the last 5 days of the experiment (sampling period), feed intake was recorded daily by weighing each animal’s offered diets and refusals after morning and evening feeding. The apparent nutrient digestibilities were determined during the sampling days according to the method described by Ferret et al. [18], where acid-insoluble ash was used as an internal marker. Fecal grab samples were collected from the rectum of each buffalo twice daily at 6 a.m. and 6 p.m., dried at 55 °C in a forced air oven for 48 h and pooled daily for each buffalo, ground to pass a 1-mm screen using a feed mill (FZ102, Shanghai-Hong Ji instrument Co., Ltd., Shanghai, China) and stored for further chemical analysis.

#### 2.2.2. Chemical Analysis

Thawed TMR samples were dried at 55 °C for 48 h, milled to pass through a 1-mm screen (FZ102, Shanghai-Hong Ji instrument Co., Ltd., Shanghai, China), and composited by treatment before chemical analysis. Samples were analyzed for analytical DM (method no. 934.01), ash (method no. 942.05), crude protein (CP; method no. 954.01), and ether extract [EE; method no. 920.39); [19]. Neutral detergent fiber (NDF; Van Soest et al. [20] and acid detergent fiber (ADF; AOAC [19]; method 973.18) analyses were conducted using an ANKOM200 Fiber Analyzer unit (ANKOM Technology Corporation, Macedon, NY, USA). For NDF assays, samples were pretreated with an α-amylase and sodium sulfite. Both NDF and ADF are expressed without residual ash, and the organic matter (OM) was calculated.

Quantitative amino acid measurements were performed for the milk protein. According to Millipore Corporation, the amino acid composition of experimental samples was determined using the HPLC-Pico-Tag method following the preparation procedure [21]. Phenyl isothiocyanate (PITC, or Edman’s reagent) was used for pre-column derivatization, while reversed-phase gradient elution high-performance liquid chromatography (HPLC) separates the phenylthiocarbamide (PTC) derivatives which were detected by their UV absorbance. The sample corresponding to the protein was weighed into a 25 × 150 mm hydrolyzed tube and was placed in a 110 °C oven for 24 h. The tube contents were quantitatively transferred to a volumetric flask and completed to volume with HPLC grade water. About 1 mL of the solution was filtered through a 0.45-µm sample filter. Together with appropriate standards, aliquots of hydrolysate were placed in disposable glass sample tubes from Waters Associates, soda glass from Fisons), loaded into a reaction vial. Hydrochloric acid was removed from the samples by drying under vacuum in a PICO-Tag workstation, achieved by connecting the vial to the workstation manifold and opening the vacuum control valve. Samples were then redried from redrying reagent (Waters reagents) again using the workstation. Derivatization is initiated by adding freshly prepared reagent mixed using a vortex mixer and allowed to stand at room temperature for 20 min. In this dried state, derivatized samples may be stored at freezer temperatures for several weeks, if required, before analysis. The chromatographic analysis using HPLC was carried out using the following gradient of Pico-Tag solvent (Eluent A and B) at 38 °C, flow rate 1 mL/min, and 20 mL of sample was injected and loaded on amino acids C18 column (100 × 4.6 mm) stainless steel. Detection of the PTC derivatives is by ultraviolet absorption measurements using a fixed wavelength (254 nm) Waters detector. Before injecting the sample, the illustrated was calibrated by two injections of the lysine standards.

Samples of fatty acid composition in milk and dried ground feed were analyzed following the method described in [22]. Briefly, 3 mL of 2 M NaOH was added to 500 mg and 100 mg of milk and feed samples, respectively, for hydrolysis of the samples in a closed system using 15-mL screw-cap Teflon-stoppered Pyrex tubes. The hydrolyzed samples were incubated in a block heater at 90 °C for 40 min. Then, samples were extracted and esterified using 0.5 M NaOH in methanol and converted to FA methyl esters (FAME) using boron trifluoride (1.3 M; Fluka-Sigma Aldrich, St. Louis, MO, USA). A gas GC-MS system (7890B, Agilent, Santa Clara, CA, USA) equipped with a 100 m fused silica capillary column (0.25 mm i.d.; coated with 0.25 μm Agilent HP; Chrompack CP7420; Agilent Technologies, Santa Clara, CA, USA) and mass spectrometer detector (5977A). Hydrogen at a flow rate of 1.3 mL/min was used as the carrier gas. The injector and detector temperatures were 200 and 250 °C, respectively. The oven temperature was programmed as follows: initially 120 °C for 7 min, then increased by 7 °C per min to 140 °C, where it was held for 10 min before being increased by 4 °C per min to 240 °C. A 1-μL sample was injected into the GC column. The peaks were identified by comparison with the retention times of appropriate FAME standards (37 FAME Mix, Sigma Aldrich, PA, USA).

Moreover, the conjugated linoleic acid peaks were identified by comparison with the retention times of a reference standard (a mixture of cis- and trans- 9,11 and 10,12-octadecadienoic acid methyl esters; Sigma Aldrich, PA, USA) using Galaxie Workstation 10.1 (Varian, CA, USA). Fatty acid compositions were expressed as g/100 g total FA. Both amino acid and fatty acid analyses were conducted at the Central Service Unit, National Research Centre, Egypt.

### 2.3. Statistical Analysis

All collected data were averaged by buffalo before chemical analysis. All parameter data (intake, digestibility, milk production/composition, milk AA, and FA profile) were analyzed using a model that included the fixed effect of treatment and the random effect of the buffalo within the treatment using PROC MIXED procedure of SAS (SAS^®^ OnDemand for Academics, 2021 SAS Institute Inc., Cary, NC, USA). The sums of squares for treatment effects were further separated into a single degree of freedom comparisons to test for the significance of preplanned contrasts as follows: (1) supporting the total mixed ration CON diet with cellulase enzymes ENZ through supplementation (CON vs. FENZ + CENZ) and (2) the source of enzyme used as a supplement FCENZ (FENZ vs. CENZ). Treatment effects were considered significant or tending towards significance at *p* ≤ 0.05 and 0.05 < *p* ≤ 0.10, respectively.

## 3. Results

The total mixed ration (TMR) offered to the lactating buffaloes (Table 1) could be characterized by a high forage content. High fibrous constitution and moderate fatty acid profile. The diet components were chosen based on; the season availability (the experiment was conducted in the middle of spring during May and June 2021), the richness in fibrous content, and was set to cover the maintenance and production requirement following the NRC (2001) manual.

### 3.1. Nutrient Intake and Digestibility

The nutrient intake and nutrient digestibility of buffaloes in the control group (CON), commercial source of cellulase (CENZ) group, and the in-farm source of cellulase (FENZ) group are shown in Table 2. Supplementing buffaloes’ diets with cellulase enzymes (CON vs. ENZ) did not significantly affect nutrient intake; no differences were observed between the cellulase enzymes sources (CFENZ). On the other hand, significant changes were observed in nutrient digestibilities. The dry matter digestibility was higher when supplementing cellulase enzymes (CON vs. ENZ). It was also significantly higher with FENZ when compared with CENZ. The same observation was applicable in the case of the digestibility of crude protein (CP), ether extract (EE), neutral detergent fiber (NDF), and acid detergent fiber (ADF), which were significantly higher with cellulase enzymes addition compared to the control (CON vs. ENZ). Supplementing the diet with an in-farm source of cellulase (FENZ) had a significantly higher impact on CP, NDF, and ADF digestibilities. However, FENZ tended to increase the EE digestibility compared to CENZ (CFENZ). Generally, FENZ showed higher nutrient digestibility percentages compared to other groups.

### 3.2. Milk Production and Composition

The milk production performance of dairy buffaloes is fully stated in Table 3. Supplementing the diet with cellulase enzymes (CON vs. ENZ) led to a significant increase in the daily milk yield and the fat correct milk yield (FCM); both yields were significantly higher with FENZ compared to all groups. Buffaloes’ diets supplemented with any source of cellulase enzyme had significantly higher fat, total solids, and solids not fat (SNF) percentages compared to control (CON vs. ENZ), it also resulted in a significantly higher fat, crude protein, and lactose yields. Repeatedly, the FENZ group resulted in significantly higher fat percentage and yield, and it is noteworthy that the highest crude protein, lactose, and energy yields were found in the FENZ group.

### 3.3. Milk Amino Acid Profile

The milk amino acids (AA) composition in lactating buffaloes is shown in Table 4. Amino acids profiles in both the essential amino acids (EAA) group and non-essential amino acids (NEAA) group were not significantly affected by cellulase enzymes supplementations (CON vs. ENZ). There were no significant differences between the cellulase enzyme sources (CFENZ) on the amino acids profile. However, enzyme supplementation showed significantly greater total EAA compared to control (CON vs. ENZ) and tended to be higher with FENZ compared to CENZ.

### 3.4. Milk Fatty Acid Profile

The milk fatty acids (FA) composition in lactating buffaloes is shown in Table 5. The milk fat concentration of C16:1 *cis*-9 and C18:0 was significantly lower with enzyme supplementation compared to the control (CON vs. ENZ). As for the C18 unsaturated fatty acids (oleic, linoleic, and linolenic acids), their concentration was significantly higher with cellulase enzymes supplementation (CON vs. ENZ) as well as the profile of the conjugated linoleic acid (*cis*-9, *trans*-11 CLA and *trans*-10, and *cis*-12 CLA). The response of supplementing lactating buffaloes’ diets with cellulase enzymes (CON vs. ENZ) was significantly higher on the sum of unsaturated fatty acids (UFA), the sum of monounsaturated fatty acids (MUFA), and the sum of polyunsaturated fatty acids (PUFA) which increased compared to CON. As well, the sum of saturated fatty acids (SFA) showed to be significantly lower when adding cellulase enzymes to buffaloes diets (CON vs. ENZ). The milk fat C10:0 tended to increase by FENZ group, while C14:0 tended to decrease by FENZ group (CFENZ), significantly, FENZ showed a lower C16:1 *cis*-9, linolenic acid, and PUFA profiles, and a higher oleic and MUFA profiles compared to the commercial source of cellulase (CFENZ).

## 4. Discussion

### 4.1. Nutrient Intake and Digestibility

Following the obtained results, the nutrient intake was not affected by cellulase supplementations, which, according to several pieces of literature [4,23,24], cellulolytic enzymes could have a marginal effect on feed intake. Consequently, that could suggest that cellulase enzymes positively affect feed intake only under suboptimal feed digestion conditions. Additionally, the amount of enzyme supplementation in this study was lower than those reported in previous studies (Peters et al. [4]; Bhasker et al. [25]), which also could explain the lack of response in nutrient intake due to an insufficient supply of enzyme activity [9]. On the other hand, the significant increase in nutrient digestibility was consistent with results obtained by [26]. That could be explained by: the increase of NDF digestibility, potential variations in gut viscosity, modified ruminal fermentation, enhanced attachment, colonization of the plant cell wall by ruminal microorganisms, and complementary actions with ruminal enzyme are possible causes of higher nutrient digestibility [27]. The increase in nutrient digestibility could also be explained by a possible synergistic effect between exogenous enzymes and endogenous enzymes, which could act as a condition modulator that increases the number of fibrolytic and non-fibrolytic microorganisms in the rumen [28].

### 4.2. Milk Production, Composition, AA and FA Profile

The increased milk yield and fat correct milk (FCM) were similarly reported by other studies using exogenous fibrolytic enzymes [4,23]. The study by Ortiz-Rodea et al. [29] conducted a large experiment on the effect of fibrolytic enzymes on milk production and composition (29 experiments). They suggested that an increase in milk and FCM yields could be encountered because of improvement in nutrient utilization and digestibility. The increase in fat content and yield due to cellulase enzyme supplementation may be credited to the substantial quantity of fiber digested in the rumen, contributing to more acetate for fatty acid synthesis [24]. The lack of effect on milk crude protein was also reported by Ortiz-Rodea et al. [29] and Peters et al. [23], consistent with a lack of differences in nutrient intake by enzymes supplements.

Based on the obtained results, only the total EAA was affected by the cellulase enzyme supplementation, which is in line with results reported by Morsy et al. [24], where high essential amino acid concentration was reached due to the presence of protease enzyme in the supplemented cocktail of enzymes. In our case, the increase of total EAA could only be due to a slight improvement of endogenous protease enzyme due to the better conditions set by the synergistic effect of cellulases.

As for the fatty acids’ composition, Rojo et al. [28] stated an increase in monounsaturated fatty acids and a lower profile of saturated fatty acids. Most milk fatty acids originated from plasma or the de novo synthesis in the mammary gland from acetate produced from rumen fermentation, including acetyl CoA carboxylase enzymes and fatty acid synthetase [24]. Ruminants do not synthesize polyunsaturated acids; consequently, their intensity in milk depends on the amount absorbed from the intestines. These results in the present study may be due to the altered contents of acetic and propionic acid production in the rumen due to better fiber digestion. The direct result of shifted VFA proportions could elevate precursor availability for fatty acid synthesis [23,29]. In our study, the conjugated linoleic acid concentration was also increased by cellulase addition, significantly, without increasing the stearic acid concentration, suggesting a slower biohydrogenation process that preserved a higher proportion of CLA without increasing stearic acid or decreasing the sum of unsaturated fatty acids [22,24]. Suggestively, the enzyme produced in the farm (FENZ) based on solid stat fermentation media is harboring multiple cellulases and hemicellulases with different specific functions that are produced in the presence of lignocellulose-based materials, which may explain the significant impact on lactating buffaloes’ performance compared to the commercial enzyme despite the equal supplemented concentration. It is also worth mentioning that the cost of producing 1 kg of farm-based cellulase enzyme cocktail cost 50 Egyptian pounds, which is equal to 0.3 Egyptian pounds for each kg of milk/day, on the other hand, the cost of 1 kg of the commercial enzyme is 300 Egyptian pound, which is equal to 1.16 Egyptian pound for each kg of milk/day.

## 5. Conclusions

The cellulase enzyme produced from *Penicillium chrysogenum* had a better impact on nutrient digestibility when supplemented with lactating buffaloes’ diets. Additionally, compared to the commercial enzyme source and the control, the produced cellulase enzyme had a better impact on the milk productivity, fat yield, and fatty acid profile. That is why we recommend establishing a small pilot production unit for cellulase enzymes in Egyptian farms, which will provide entrepreneurs with small farms to benefit from agricultural and agro-industrial wastes located near the farm.

## Figures and Tables

**Table 1:** Ingredients and chemical composition of the total mixed ration (TMR).

Item	TMR
Ingredients, g/kg of DM	
Corn grain	52.0
Soybean meal	112
Wheat bran	180
Sunflower meal	43.5
Berseem hay	350
Beet pulp	150
Rice straw	100
Mineral-vitamin mixture	12.5
Chemical composition, g/kg of DM	
Organic matter	908
Ash	92.0
Crude Protein	168
Ether Extract	41.0
Neutral detergent fibre	385
Acid detergent fibre	219
Fatty acid composition, g/100g of FA	
C14:0	0.92
C16:0	26.3
C18:0	5.93
C18:1 cis-9	16.9
C18:2 cis-9 cis-12	38.6
C18:3 cis-9 cis-12 cis-15	9.83

**Table 2:** Nutrient intake and digestibility of dairy buffaloes as affected by cellulase enzyme supplementation to the diet.

Item		Treatments ^1^		SEM	Contrast ^2^	
	CON	CENZ	FENZ		CON vs. ENZ	CFENZ
Nutrient Intake, kg/d						
Dry matter	15.9	15.7	15.7	1.404	0.347	0.886
Organic matter	14.2	13.9	14.1	1.278	0.158	0.745
Crude protein	2.67	2.63	2.64	0.266	0.114	0.623
Either extract	0.65	0.64	0.65	0.100	0.202	0.668
Neutral detergent fibre	6.12	6.03	6.06	0.402	0.234	0.569
Acid detergent fibre	3.48	3.43	3.45	0.527	0.161	0.421
Nutrient Digestibility, %						
Dry matter	60.2	63.2	66.2	1.104	0.016	0.033
Organic matter	59.5	62.5	65.4	1.277	0.008	0.021
Crude protein	60.5	63.5	66.5	1.603	0.004	0.022
Either extract	59.9	62.9	63.9	2.285	0.024	0.059
Neutral detergent fibre	53.2	55.9	58.5	0.586	0.013	0.016
Acid detergent fibre	55.3	58.1	60.8	2.458	0.029	0.039

^1^ Treatments: CON—control diet consisted of total mixed ration, CENZ—control diet supplemented with a commercial source of cellulase enzyme, FENZ—control diet supplemented with cellulase enzyme produced in the farm; ^2^ Significance of effects due to supplementing buffaloes’ diet with cellulase enzyme (CON vs. ENZ; CON vs. CENZ + FENZ) and source of cellulase enzyme supplemented to the diet (CFENZ; CENZ vs. FENZ).

**Table 3:** Milk production performance of dairy buffaloes as affected by cellulase enzyme supplementation to the diet.

Item	Treatments ^1^			SEM	Contrast ^2^	
	CON	CENZ	FENZ		CON vs. ENZ	CFENZ
Milk production (kg/d)						
Milk yield	7.90	8.30	8.69	0.223	0.028	0.041
3.5% FCM ^3^	11.8	12.8	13.9	0.421	0.014	0.019
Milk composition, %						
Fat	6.52	6.85	7.19	0.023	0.038	0.022
Crude protein	3.98	4.03	4.11	0.012	0.286	0.523
Lactose	4.61	4.71	4.66	0.018	0.344	0.644
Total Solids	15.9	16.4	16.8	0.561	0.019	0.112
SNF ^4^	9.44	9.59	9.62	0.188	0.018	0.687
Energy (Mcal/Kg)	1.01	1.05	1.08	0.001	0.659	0.705
Milk yields (g/day)						
Fat	515	568	625	1.662	0.008	0.012
Crude protein	314	334	357	1.521	0.001	0.022
Lactose	364	391	405	1.592	0.011	0.023
Energy (Mcal)	7.97	8.68	9.38	0.327	0.020	0.003

^1^ Treatments: CON—control diet consisted of total mixed ration, CENZ—control diet supplemented with a commercial source of cellulase enzyme, FENZ—control diet supplemented with cellulase enzyme produced in the farm; ^2^ Significance of effects due to supplementing buffaloes’ diet with cellulase enzyme (CON vs. ENZ; CON vs. CENZ + FENZ) and source of cellulase enzyme supplemented to the diet (CFENZ; CENZ vs. FENZ); ^3^ 3.5% fat-corrected milk; ^4^ Solid not fat.

**Table 4:** Milk amino acids composition (g/100 g of milk protein) of dairy buffaloes as affected by cellulase enzyme supplementation to the diet.

Item	Treatments ^1^			SEM	Contrast ^2^	
	CON	CENZ	FENZ		CON vs. ENZ	CFENZ
Essential Amino Acids (EAA)						
Arginine	2.30	2.07	1.84	0.053	0.620	0.156
Histidine	2.15	2.18	2.24	0.082	0.767	0.692
Isoleucine	4.62	4.78	4.68	0.048	0.691	0.855
Leucine	8.30	9.62	9.82	0.051	0.217	0.743
Lysine	6.60	6.89	6.52	0.058	0.058	0.066
Methionine	7.39	8.42	8.15	0.053	0.106	0.644
Phenylalanine	3.61	3.61	4.03	0.089	0.648	0.532
Threonine	4.07	4.46	5.32	0.068	0.138	0.522
Valine	5.28	5.67	5.61	0.078	0.727	0.698
Total EAA	44.3	47.7	48.2	0.835	0.031	0.069
Non-Essential Amino Acids (NEAA)						
Alanine	3.35	3.15	3.62	0.112	0.728	0.779
Aspartic acid	6.71	6.87	7.06	0.076	0.412	0.685
Glutamic acid	20.7	20.2	19.9	0.032	0.769	0.532
Serine	4.18	4.56	4.94	0.135	0.612	0.551
Tyrosine	2.52	2.83	2.42	0.111	0.388	0.632
Total NEAA	37.5	37.6	37.9	3.927	0.684	0.895

^1^ Treatments: CON—control diet consisted of total mixed ration, CENZ—control diet supplemented with a commercial source of cellulase enzyme, FENZ—control diet supplemented with cellulase enzyme produced in the farm; ^2^ Significance of effects due to supplementing buffaloes’ diet with cellulase enzyme (CON vs. ENZ; CON vs. CENZ + FENZ) and source of cellulase enzyme supplemented to the diet (CFENZ; CENZ vs. FENZ).

**Table 5:** Milk fatty acid composition (g/100 g of total FA) of dairy buffaloes as affected by cellulase enzyme supplementation to the diet.

Item	Treatments ^1^			SEM	Contrast ^2^	
	CON	CENZ	FENZ		CON vs. ENZ	CFENZ
C8:0	1.79	1.81	1.76	0.031	0.829	0.192
C10:0	1.56	1.43	1.52	0.066	0.757	0.059
C12:0	2.01	1.79	1.92	0.071	0.484	0.096
C14:0	11.5	11.5	10.5	0.649	0.790	0.067
C14:1 cis-9	0.71	0.73	0.73	0.016	0.851	0.756
C16:0	32.9	31.0	32.5	0.959	0.459	0.264
C16:1 cis-9	1.35	1.36	1.06	0.091	0.041	0.001
C18:0	17.6	16.9	16.2	0.557	0.035	0.144
C18:1 trans-10	0.28	0.27	0.28	0.015	0.916	0.788
C18:1 trans-11	1.14	1.42	1.26	0.085	0.421	0.325
C18:1 cis-9	21.9	23.5	25.6	1.113	0.019	0.022
C18:2 cis-9 cis-12	1.56	1.89	1.88	0.019	0.019	0.242
C18:3 cis-9 cis-12 cis-15	0.67	0.99	0.88	0.090	0.022	0.033
C18:2 cis-9 trans-11	0.40	0.64	0.63	0.008	0.012	0.346
C18:2 trans-10 cis-12	0.12	0.16	0.15	0.005	0.036	0.621
C20:0	0.73	0.55	0.79	0.015	0.002	0.001
C22:1	0.16	0.26	0.22	0.005	0.018	0.211
Other FA ^3^	4.53	3.79	2.09	0.122	0.006	0.018
SFA ^4^	68.1	65.1	65.2	2.356	0.013	0.452
UFA ^5^	31.9	34.9	34.7	1.489	0.001	0.566
MUFA ^6^	25.6	27.5	29.1	1.023	0.026	0.031
PUFA ^7^	6.29	7.49	5.63	0.745	0.002	0.012

^1^ Treatments: CON—control diet consisted of total mixed ration, CENZ—control diet supplemented with a commercial source of cellulase enzyme, FENZ—control diet supplemented with cellulase enzyme produced in the farm; ^2^ Significance of effects due to supplementing buffaloes’ diet with cellulase enzyme (CON vs. ENZ; CON vs. CENZ + FENZ) and source of cellulase enzyme supplemented to the diet (CFENZ; CENZ vs. FENZ); ^3^ Sum of other fatty acids including C6, C10:1, C11:0, C13:0, C16:1 trans, C18:1 trans-5, C18:1 trans-6–8, C18:1 trans-9, C19:0, C18:2 cis-9, cis-15, C20:1 trans, C21:0, C20:2, C22:0, C20:3n-6, C20:3n-3, C23:0, C22:2, C24:0, and C24:1; ^4^ Sum of saturated fatty acids; ^5^ Sum of unsaturated fatty acids; ^6^ Sum of monounsaturated fatty acids; ^7^ Sum of polyunsaturated fatty acids.

## Data Availability

The data presented in this study are available on request from the corresponding author.
